# Drug Repurposing for COVID-19: A Review and a Novel Strategy to Identify New Targets and Potential Drug Candidates

**DOI:** 10.3390/molecules27092723

**Published:** 2022-04-23

**Authors:** Liliana Rodrigues, Renata Bento Cunha, Tatiana Vassilevskaia, Miguel Viveiros, Celso Cunha

**Affiliations:** Global Health and Tropical Medicine, Instituto de Higiene e Medicina Tropical, Universidade NOVA de Lisboa, Rua da Junqueira 100, 1349-008 Lisboa, Portugal; rbentocunha@ihmt.unl.pt (R.B.C.); tatianav@ihmt.unl.pt (T.V.); mviveiros@ihmt.unl.pt (M.V.); ccunha@ihmt.unl.pt (C.C.)

**Keywords:** SARS-CoV-2, COVID-19, drug repurposing, computer-aided drug discovery

## Abstract

In December 2019, the novel severe acute respiratory syndrome coronavirus 2 (SARS-CoV-2), the causative agent of coronavirus disease 2019 (COVID-19) was first identified in the province of Wuhan, China. Since then, there have been over 400 million confirmed cases and 5.8 million deaths by COVID-19 reported worldwide. The urgent need for therapies against SARS-CoV-2 led researchers to use drug repurposing approaches. This strategy allows the reduction in risks, time, and costs associated with drug development. In many cases, a repurposed drug can enter directly to preclinical testing and clinical trials, thus accelerating the whole drug discovery process. In this work, we will give a general overview of the main developments in COVID-19 treatment, focusing on the contribution of the drug repurposing paradigm to find effective drugs against this disease. Finally, we will present our findings using a new drug repurposing strategy that identified 11 compounds that may be potentially effective against COVID-19. To our knowledge, seven of these drugs have never been tested against SARS-CoV-2 and are potential candidates for in vitro and in vivo studies to evaluate their effectiveness in COVID-19 treatment.

## 1. Introduction

The emergence of the novel severe acute respiratory syndrome coronavirus 2 (SARS-CoV-2) created a worldwide public health emergency. SARS-CoV-2 is the causative agent of coronavirus disease 2019 (COVID-19) and was first isolated during an outbreak of SARS in the province of Wuhan, China, in December 2019 [1]. Due to its rapid dissemination to several countries and virtually all continents, the World Health Organization (WHO) declared a pandemic in March 2020 and there have been over 400 million confirmed cases and 5.8 million deaths of COVID-19 reported worldwide until February 2022 [2]. At present, the situation is still vastly uncontrolled in many parts of the world.

Coronaviruses are large, enveloped, positive-sense, single-stranded RNA viruses found to infect different animal species, including reptiles, birds, and mammals. It has been widely reported that coronaviruses, similarly to other viruses, can occasionally “jump” between species and adapt to the new host. The Middle East respiratory syndrome coronavirus (MERS-CoV) and the severe acute respiratory syndrome coronavirus (SARS-CoV) are recent examples of the adaptation to infect and replicate in humans of coronaviruses previously thought to be confined to their natural host reservoirs—bats. However, zoonotic transmission of MERS and SARS-CoV to humans was not direct but rather involved as intermediate hosts, dromedary camels and civets, respectively. Similarly to MERS-CoV and SARS-CoV, SARS-CoV-2 perhaps also had a zoonotic origin. It is probably that it also originated in bats, but an intermediate host could not still be unequivocally identified. SARS-CoV-2 is primarily transmitted through direct contact with respiratory virus-containing droplets and aerosols from infected individuals. Coughing, sneezing, and nasal discharge are important sources of contagium. However, the identification of SARS-CoV-2 genetic material in several organs points to a broad tropism, not only restricted to the upper and lower respiratory tracts. This may partly be explained by the fact that the main cellular receptor for SARS-CoV-2, angiotensin-converting enzyme 2 (ACE-2), is expressed in several human tissues and organs [3]. Most common COVID-19 symptoms include fever, cough, fatigue, and dyspnoea [4]. However, impairment of neurologic, cardiac, liver, kidney, and many other organ functions were reported in patients and convalescent individuals. Disease outcome is not rarely fatal, most commonly due to severe viral pneumonia symptoms affecting especially elder people and immunosuppressed individuals. Moreover, the presence of other concomitant clinical conditions, such as chronic cardiac disease and diabetes, are also thought to represent important risk factors affecting disease outcome.

Humans are infected regularly and worldwide with the so-called seasonal coronaviruses, which usually cause a respiratory disease with mild symptoms. They are not recognized as an important public health threat, and development of a specific anti-viral treatment or preventive vaccine was not considered a priority. Therefore, when SARS-CoV-2 emerged, there were no specific antiviral treatments for coronavirus diseases, including COVID-19. Classical approaches to identify new specific anti-viral compounds as well as development of new therapeutic options is a long and complex process that may take several years. In this context, drug repositioning has emerged as a promising and potentially useful approach to identify already approved drugs for treatment of other diseases, including COVID-19 [5]. The main advantages of drug repositioning include the availability of information about pharmacokinetics, pharmacodynamics, and toxicity, of a given drug of interest [6]. Using similar strategies to search for anti-SARS-CoV-2 compounds may significantly shorten the time needed to find an effective treatment for COVID-19, reducing disease burden, including number of hospital admissions, deaths, and long-term sequelae.

Since the beginning of the pandemic, several potential anti-SARS-CoV-2 drugs have been under investigation in clinical trials, including remdesivir (initially developed by Gilead for Hepatitis C treatment), chloroquine and hydroxychloroquine (well-known drugs used in malaria treatment), tocilizumab (a monoclonal antibody used in rheumatoid arthritis), favipiravir (an anti-influenza drug), Kaletra (used for HIV treatment), and more recently masitinib (a kinase inhibitor used for mast cell tumour treatment in animals) [7]. More recently, the EU approved the emergency use of Lagevrio (also known as molnupiravir developed by Merck) and Paxlovid (Pfizer) to treat adults with COVID-19 who do not require supplemental oxygen and who are at increased risk of developing severe COVID-19 [8].

Here, we will focus on developments in state-of-the-art research concerning drug repurposing for COVID-19 treatment. SARS-CoV-2 specific factors that are known to interact with host cellular machinery and represent potential targets for drug repositioning will be discussed. Finally, we will present our findings using a drug repurposing strategy to identify new anti-SARS-CoV-2 compounds that may be potentially effective in COVID-19 treatment.

## 2. Key Targets for Drug Development

Similarly to other coronaviruses, the SARS-CoV-2 RNA genome encodes four major structural proteins: spike (S), nucleocapsid (N), membrane (M), and envelope (E). In addition, the virus genome encodes sixteen non-structural proteins (Nsp 1–16), all of which are required during different steps of the virus replication cycle [4,9]. These proteins interact with the host cellular machinery for virus replication and production of new virions. As an example, viral entry into cells occurs via clathrin-mediated endocytosis after interaction of SARS-CoV-2 S protein with the cellular ACE2 receptor [3]. Theoretically, any SARS-CoV-2 encoded protein may represent a valid and potential therapeutic target.

### 2.1. SARS-CoV-2 Drug Targets

SARS-CoV-2 structural proteins play different roles in virus replication. The S protein is responsible for attachment to the cell receptor playing a role in internalization of the virus, also defining host-tissue tropism, and viral transmission capacity [10]. It is composed of two functional subunits, S1 and S2. Subunit S1 is responsible for binding to host cell receptor ACE-2 and contains two identified domains: N-terminal domain (NTD) and receptor binding domain (RBD). Subunit S2 is responsible for fusion of viral and cellular membranes and contains seven domains: fusion peptide (FP), heptad repeat 1 (HR1), central helix (CH), connector domain (CD), heptad repeat 2 (HR2), transmembrane domain (TM), and cytoplasmic tail (CT) [9,10]. The cleavage site at the border between S1 and S2 subunits is called S1/S2 protease cleavage site [10]. Some host proteases were found to promote this cleavage, namely type II transmembrane serine protease (TMPRSS2) and furin. Cleavage at the S1/S2 border promotes viral entry into host cells.

The E protein is the smallest of the major structural proteins and is part of the lipid-containing envelope of SARS-CoV-2. It has a well-established role in assembly of virions, envelope formation, budding, and pathogenesis. Along with its interaction with M protein for viral envelope assembly, E protein also interacts with accessory proteins and host cell proteins. More recent evidence seems to indicate that changes in E protein stability may affect viral conformation and functional processes, potentially affecting pathogenesis of SARS-CoV-2 [11]. However, further studies are needed to confirm this hypothesis.

The N protein is the most abundant protein expressed by SARS-CoV-2 in infected cells. It plays a crucial role in packaging the viral RNA genome into long, flexible, helical ribonucleoprotein (RNP) complexes [12,13]. Three distinct and highly conserved domains were identified in this protein: a N-terminal domain (NTD), a C-terminal domain (CTD) and an RNA-binding domain (RBD). In addition, the N protein plays an essential structural role through a network of interactions with virus genomic RNA, M protein, and making complexes with other N protein molecules [13].

The M protein is the most abundant protein in the virion envelope and has three transmembrane domains and a large carboxyl-terminal tail. It plays a fundamental role in virion assembly and might also display ion channel activity [12,14]. In addition, recent evidence suggests that M protein, in association with N protein, may play a role in assembly of the nucleocapsid core into progeny virions [14].

The sixteen non-structural proteins are encoded by two ORFs, 1a and 1b, and originate by specific cleavage events of either polyprotein 1a or polyprotein 1b [12]. They play diverse important roles in the virus replication cycle, and their functions are summarized in Table 1.

The deeper understanding and knowledge of structure, function, and cellular targets of SARS-CoV-2 encoded proteins may allow developing and repurposing molecules capable of interfering with their functions. Ultimately, this may lead to development of a potential specific and effective treatment for COVID-19.

### 2.2. Host-Based Drug Targets

An increasing body of evidence supports a paradigm where virus-interacting host molecules are expected to represent the next frontier in antiviral drug discovery. These cellular proteins are essential players in modulation of virus replication. Targeting host proteins has obvious advantages over targeting virus proteins. One of the main reasons is the widely reported loss of efficacy of antiviral agents due to the high mutation rate of viral genomes that may ultimately potentiate the emergence and selection of new resistant variants. However, there are also some important drawbacks. One of them is the fact that many host functions targeted by drugs, are essential for maintaining proper cell function and homeostasis. As consequence, severe toxic effects are often associated with administration of this type of compounds.

Entry of SARS-CoV-2 into cells involves the attachment of S protein to the ACE2 receptor located at the cell surface. This process facilitates viral entry and is associated with subtle conformational changes [3]. After SARS-CoV-2 interaction with the ACE2 receptor, host type II transmembrane protease serine 2 (TMPRSS2) catalyses the cleavage of SARS-CoV-2 spike protein triggering viral entry [27]. Thus, both Spike/ACE2 interaction and TMPRSS2 cleavage activity may represent important potential targets for antiviral intervention.

Another important step in SARS-CoV-2 entry into host cells involves clathrin-mediated endocytosis (CME) [28]. In fact, recent evidence suggests that CME is also crucial for viral entry since clathrin-heavy chain knockdown significantly reduces viral infectivity [10,28].

Bayati and co-workers have recently proposed a viral infectivity model for SARS-CoV-2 involving three key steps: (1) viral S protein binds to the plasma membrane of cells expressing the ACE2 receptor; (2) ACE2/virus complex undergoes rapid clathrin-mediated endocytosis with virus delivery to the lumen of the endosome; (3) viral envelope and lumen of the endosomal membrane fuse, promoting viral RNA release into the cytosol and subsequent steps of the replication cycle [28]. Blocking any of these steps may help preventing virus infection, and consequently may also represent valid starting targets for drug repositioning.

Besides SARS-CoV-2 interaction with host factors required for entry, the virus also interacts with factors required for viral RNA synthesis and virion assembly (such as the endoplasmic reticulum (ER), Golgi components and associated vesicular trafficking pathways), as well as factors required for translation of viral mRNA [29]. In fact, screening studies have identified various proteins localizing to the ER, the ER-Golgi intermediate compartment, and the Golgi apparatus as critical for virus replication highlighting the roles of these compartments in translation of SARS-CoV-2 proteins and further virion assembly [30].

## 3. Pharmacological Approaches against COVID-19

Even with effective vaccines already available, it is essential to identify and develop new drugs for COVID-19 treatment in order to decrease the burden of disease, including the number of hospital stays and mortality, especially in high-risk groups. Multiple efforts are being made to achieve this goal. On June 2021, the Biden administration allocated more than USD 3 billion to accelerate the discovery and development of the next generation of COVID-19 treatments. As of December 2021, there were 2019 drug studies listed in Clinicaltrials.gov and 655 mapped drug names [7].

At present, COVID-19 patient management is still largely focused on relief of symptoms and respiratory support. In patients presenting moderate to severe COVID-19 symptoms, several drugs have been tested including antiviral compounds, inflammation inhibitors, low molecular weight heparins, and plasma immunoglobulins [31]. In most cases, these drugs were already approved to treat other conditions, a strategy known as drug repurposing or repositioning. It is estimated that development of a new pharmacologically active drug costs, on average, approximately USD 1.24 billion. This includes the whole process from traditional drug discovery to market introduction. In the case of drug repurposing, this process is estimated to be at least 40% less expensive [32,33]. Moreover, it usually takes 10–16 years to develop a new drug using the traditional drug discovery approach, while an average of 8 years is needed to develop a repurposed drug. In fact, a repurposed drug can enter directly to preclinical testing and clinical trials, thus reducing the risk, time, and costs associated with drug development [32,33]. Next, we conduct an overview of different repurposed agents that have been considered for COVID-19 treatment (Table 2). Unfortunately, some of them did not show benefits for patients, but others are still in use albeit with variable efficacy. We focused on the most relevant compounds tested until now, including antiviral and antiparasitic drugs, steroids, signalling inhibitors, and monoclonal antibodies, albeit new compounds should be added to this list as new evidence arises on the mechanism of infection of SARS-CoV-2 and new pharmacological approaches are envisaged to control it.

### 3.1. Antiparasitic Drugs

#### 3.1.1. Chloroquine and Hydroxychloroquine

Chloroquine is an antimalarial agent with anti-inflammatory and immunomodulatory activities that has attracted interest as a potential therapeutic option for treatment of COVID-19-associated pneumonia [34,35,36,37]. Hydroxychloroquine, chloroquine’s hydroxylated form, displays a similar mechanism of action but has shown better tolerability allowing its use in long-term treatment of rheumatological disorders [38,39]. Both compounds have been used mainly in prevention and treatment of malaria and chronic inflammatory diseases such as systemic lupus erythematosus and rheumatoid arthritis [38,39,40]. However, they have also shown a broad antiviral activity against HIV, SARS-CoV, Marburg, Zika, Dengue, and Ebola viruses. This fact raised the interest of the scientific community to explore their use as anti-SARS-CoV-2 agents in the treatment of COVID-19 [36,37]. Previous studies have shown that chloroquine and hydroxychloroquine interfere with virus entry and decapsidation due to its ability to modulate endosomal pH, interfere with glycosylation status of host ACE2 receptor, and compete with the spike protein in binding to gangliosides [37,40]. Moreover, both compounds were shown to modulate the immune system by affecting cell signalling and the production of proinflammatory cytokines [37,41]. These features made hydroxychloroquine an attractive and commonly used repurposed drug during the initial phase of the COVID-19 pandemic. Several clinical trials with these drugs have been carried out since then, but the results were disappointing. In fact, they have shown a lack of efficacy in both postexposure prophylaxis and treatment of mild/moderate COVID-19, as well as inability to reduce mortality rates when compared to the standard care supporting treatment. Additionally, severe side effects were observed, namely relating to cardiovascular problems (QT prolongation with fatal arrhythmias), liver or kidney damage, retinopathy, and hypoglycaemia [36,37,42,43,44]. The absence of clearly proved benefits coupled with safety concerns has contributed to the decision of the WHO to discontinue hydroxychloroquine clinical trials for treatment of COVID-19 patients [43].

#### 3.1.2. Ivermectin

Ivermectin is a broad spectrum anti-parasitic agent approved by the Food and Drug Administration (FDA) as an oral treatment for intestinal strongyloidiasis and onchocerciasis and as a topical treatment for pediculosis and rosacea [45]. It was suggested that the activity of ivermectin against SARS-CoV-2 might be due to the inhibition of host importin α/β-mediated nuclear transport of proteins [46]. Previous studies have demonstrated that ivermectin may decrease SARS-CoV-2 replication in vitro but mostly at higher concentrations than those reached with the authorised doses [47,48]. Usually, ivermectin is well tolerated at therapeutic doses for anti-parasitic treatment, but side effects could increase with the much higher concentrations that would be needed for this drug to be effective against SARS-CoV-2. Moreover, the current evidence from clinical trials on the use of ivermectin to treat COVID-19 patients is inconclusive, with some studies showing no benefit and others reporting a potential benefit in the prevention or treatment of COVID-19. In fact, the WHO recommends the use of ivermectin only within clinical trials.

### 3.2. Signalling Inhibitors

#### 3.2.1. Baricitinib

Baricitinib is a small molecule inhibitor of the Janus-associated kinases 1 and 2 (JAK1 and JAK2), approved for treatment of rheumatoid arthritis [36,49,50]. Baricitinib was selected as a potential repurposed drug against SARS-CoV-2 through an artificial intelligence algorithm. It was demonstrated that the mechanism of action against COVID-19 relies on the modulation of the cytokine storm caused by the infection as well as the inhibition of virus entry into host cells [49]. Clinical trials have shown that baricitinib in combination with remdesivir was more effective than remdesivir alone in reducing recovery time in COVID-19 patients receiving high-flow oxygen or non-invasive ventilation [51]. The emergency use of baricitinib in combination with remdesivir for treatment of hospitalized COVID-19 patients requiring oxygen or mechanical ventilation was authorized by the FDA in November 2020 [52]. Later, in July 2021, the FDA revised the emergency use authorization for baricitinib, now authorizing its use alone, without remdesivir [53]. On April 2021, the European Medicines Agency (EMA) started assessing the extension of indication of baricitinib for treatment of COVID-19 in hospitalised patients who require supplemental oxygen.

#### 3.2.2. Masitinib

Masitinib is a tyrosine kinase inhibitor previously evaluated for treatment of several non-communicable diseases such as cancer, asthma, Alzheimer’s disease, multiple sclerosis, and amyotrophic lateral sclerosis [54,55,56,57,58]. It has been shown that masitinib may also act as a broad antiviral agent by inhibiting the activation of virus-specific proteins, namely the proteases of two positive-strand RNA viruses, coronaviruses, and picornaviruses. This compound was shown to be effective in vitro against all SARS-CoV-2 variants of concern [59]. Experiments in mice also showed significant reduction in virus titres in lungs. Masitinib has been shown to completely inhibit the SARS-CoV-2 main protease, 3CL, essential for viral replication and well conserved among coronaviruses [60]. In September 2021, AB Science has obtained approval from the Regulatory Authorities of Russia and South Africa to commence a phase 2 clinical trial with masitinib for COVID-19 treatment.

### 3.3. Monoclonal Antibodies

#### Tocilizumab

Tolicizumab is a monoclonal antibody, approved for treatment of rheumatoid arthritis and other autoimmune rheumatic diseases such as systemic juvenile idiopathic arthritis [61]. The mechanism of action consists of binding to soluble and membrane-bound interleukin 6 (IL-6) receptors, thus preventing IL-6 activity. This cytokine plays a role in inflammation processes and its elevated production is associated with development of acute respiratory distress syndrome in COVID-19 patients [62,63]. Therefore, agents such as tocilizumab could display potential beneficial effects against COVID-19. Most clinical trials have reported that treatment with tocilizumab has reduced the mortality rate comparatively to non-treated patients and with no evidence of significant toxicity effects [64,65]. It was also shown that patients that mostly benefit from the use of tocilizumab in combination with standard care, are those presenting moderate to severe symptoms, although not yet requiring mechanical ventilation [66]. Tocilizumab, when used together with standard care, reduced the probability of progression to mechanical ventilation and death rates in hospitalized patients with COVID-19 pneumonia [66]. However, at least one study demonstrated that early administration of tocilizumab did not influence the mortality rate or time of recovery [67]. In July 2021, tocilizumab became the second drug recommended by the WHO for COVID-19 treatment (dexamethasone, an anti-inflammatory drug, was recommended in September 2020) [68].

### 3.4. Steroids

#### Dexamethasone

Dexamethasone is a glucocorticoid commonly used to treat severe allergies, asthma, several forms of arthritis and intestinal disorders. It is part of the WHO list of essential medicines [69]. This drug displays strong anti-inflammatory and immunosuppressant properties and was recommended in the second half of 2020 for treatment of COVID-19 patients who need mechanical ventilation or supplemental oxygen. Dexamethasone acts by binding to the glucocorticoid receptor triggering a signalling cascade that ultimately results in inhibition of expression of inflammatory genes and stimulation of expression of anti-inflammatory genes [70]. In COVID-19 patients, this drug acts by suppressing the overstimulation of the immune system, namely the type 1 interferon response, caused by SARS-CoV-2 infection of the lungs [71]. Dexamethasone was the first drug to display a significant impact in reducing the death rate of hospitalized patients with severe symptoms, ranging from 20% to 35% [72].

### 3.5. Antiviral Drugs

#### 3.5.1. Umifenovir

Umifenovir (Arbidol^TM^) is an indole carboxylic acid derivative that presents inhibitory activity against a variety of viruses such as parainfluenza, influenza A and B, and hepatitis C virus [73,74,75]. It was developed by Pharmastandard for the treatment of influenza and is currently used mainly in Russia and China. The mechanism of action consists of preventing the virus-cell membrane fusion and virus-endosome internalization through the incorporation of umifenovir molecules into the cell membrane [76,77]. Moreover, it was shown that umifenovir can enhance the humoral immune response and interferon production. It has been hypothesized that the combination of umifenovir with interferons may produce a synergistic therapeutic effect against SARS-CoV-2 [78]. Previous studies have also shown that the combination of umifenovir with lopinavir-ritonavir was associated with an increased RT-PCR SARS-CoV-2 negative conversion rate, when compared with treatment with lopinavir-ritonavir alone [79]. A recent randomized clinical trial showed that umifenovir did not improve mortality rates and did not decrease the need for mechanical ventilation and time to clinical improvement [80].

#### 3.5.2. Remdesivir

Remdesivir (Veklury^TM^) was originally developed by Gilead Sciences, Inc. (Foster City, CA, USA) as a potential anti-Hepatitis C virus candidate and was later repurposed and tested against Ebola and Marburg viruses in clinical trials. In both cases, its development was stopped due to the lack of solid evidence about its efficacy [81,82]. When administered, the GS-441524 monophosphate prodrug is intracellularly converted into the corresponding triphosphate that acts as adenosine ribonucleotide triphosphate analogue. It was shown to inhibit the activity of the virus-encoded RNA-dependent RNA polymerase, impairing RNA synthesis and replication [83,84]. This prodrug was thought to be a potential promising candidate to be repurposed for COVID-19 treatment since almost the beginning of the pandemic. Phase III clinical trials were carried out in early 2020. However, the efficacy of remdesivir was, in several moments, questionable and surrounded in controversy, due to the lack of evidence of its efficacy in mortality reduction. Several important adverse side effects were observed, including hepatocellular toxicity, nausea, anaemia, kidney injury, hypotension, respiratory failure, and constipation [85]. Nevertheless, remdesivir is one of the medications recommended by the EMA and the FDA for treatment of severe COVID-19 since it may reduce hospitalization time [86,87].

#### 3.5.3. Favipiravir

Favipiravir (brand name Avigan™) is a nucleoside analogue prodrug that mimics both adenosine and guanosine. It was initially developed for treatment of seasonal influenza by Toyama Chemical and is available as a therapeutic agent in some countries such as China and Japan [88]. Favipiravir displays a mechanism of action similar to that of remdesivir, acting by inhibiting viral RNA-dependent RNA polymerase activity [89,90,91]. This compound was initially used for treatment of COVID-19 in Wuhan. Since then, several clinical trials have been carried out to evaluate its efficacy against SARS-CoV-2. There is still limited data available, but there is evidence pointing to improvement of some clinical and radiological indicators. However, no reduction in mortality or differences in oxygen-support requirement were observed [92,93]. Large-cohort clinical trials began in May 2021 and are currently ongoing [94].

#### 3.5.4. Molnupiravir

Molnupiravir (Lagevrio™, Merck) was the first oral, direct-acting antiviral shown to be highly effective at reducing nasopharyngeal SARS-CoV-2 titres, and to have a significant benefit in reducing hospitalization or death in mild COVID-19 [95,96]. It is a prodrug of N4-hydroxycytidine that is converted into the active form 5′-triphosphate by host kinases. The active 5′-triphosphate serves as a competitive substrate for the viral RNA-dependent RNA polymerase and causes an antiviral effect through the accumulation of mutations after each viral replication cycle. This drug was originally developed as a possible treatment of diseases caused by RNA viruses, namely influenza viruses and encephalitic alphaviruses, but also showed a broad-spectrum antiviral activity against several coronaviruses, including SARS-CoV-2, in preclinical studies [96]. Recently, the pharmaceutical company Merck released the final analysis of a clinical trial that demonstrated that molnupiravir reduced the risk of hospitalization and death among high-risk patients by 30%, instead of the earlier estimate of 50%. EMA granted emergency use authorization of Lagevrio™ (molnupiravir) in patients who do not require supplemental oxygen and who are at increased risk of developing severe COVID-19. Furthermore, FDA also authorized the use of molnupiravir in certain adult patients.

#### 3.5.5. Lopinavir and Ritonavir

Lopinavir and ritonavir (Kaletra™, Abbott) are used for the management of AIDS. These drugs specifically inhibit one of the key factors required during the virus replication cycle, the virus-encoded protease responsible for processing newly synthesized virus polyproteins [97]. Previous studies have shown that these compounds may also be effective against MERS and SARS viruses whose genomes also code for specific proteases with similar functions [98,99]. As a consequence, they became attractive as potential anti-SARS-CoV-2 drugs. In fact, it was suggested that lopinavir-ritonavir could decrease SARS-CoV-2 replication in vitro through inhibition of the virus-specific 3CL1 proprotease [100]. However, results from subsequent multiple clinical trials were controversial regarding the capacity of these drugs to improve disease outcomes. The WHO-promoted Solidarity trials stopped testing this combination due the absence of clear benefits, including to the lack of mortality reduction in hospitalized patients [101].

#### 3.5.6. Paxlovid

A more effective treatment with Paxlovid™ (Pfizer), showed 89% efficacy in reducing hospitalization and death. Paxlovid™ consists of a combination of nirmatrelvir (PF-07321332) and ritonavir. Nirmatrelvir belongs to a group of compounds previously shown to specifically inhibit the coronavirus protease 3CL and successfully tested against SARS and Feline coronaviruses [102,103]. It cannot be considered a true repurposed drug since it was initially synthesized with the purpose to be tested against SARS-CoV-2. Nirmatrelvir acts by binding to the active site of the SARS-CoV-2 serine protease 3CL specifically inhibiting its activity. Ritonavir is another protease inhibitor commonly used for HIV/AIDS and hepatitis C treatment [104]. When administered together with ritonavir, PF-07321332 is degraded slower inside the cell, its concentrations remain higher for longer periods of time, and the efficacy is increased.

EMA authorized the use Paxlovid™ in January 2022. In addition, FDA also authorized the emergency use of Paxlovid™ for treatment of mild-to- moderate COVID-19 in certain adults and paediatric patients.

## 4. In Silico Repurposing Strategies to Identify Potential Drugs and Drug Targets against COVID-19

Presently, drug repurposing no longer depends on serendipitous observations, but is instead the result of the application of computational and biological strategies that allow the quick and effective selection of the most promising repurposed candidates [105]. In general, drug repurposing follows one of two principles: (i) drug-based, which is directed to the discovery of new uses for a particular drug; (ii) target-based, which selects targets based on their homology to a target for which a drug has already been approved.

An example of an in silico drug repurposing approach is to take advantage of the large amount of data publicly available in online databases that provide information on thousands of therapeutic drugs [106,107,108]. Thus, it is possible to identify a list of approved agents with predictive activity against SARS-CoV-2 that may be directly testable in preclinical and clinical trials for COVID-19. Moreover, the development and implementation of machine learning and artificial intelligence (AI) methods can greatly revolutionize computational drug repurposing and accelerate the discovery of anti-COVID-19 repurposed drugs [109]. Computer-aided approaches applied to drug discovery against COVID-19 have mostly employed docking screen, molecular simulation, pharmacophore models, or machine learning-based virtual screens [110]. There are several reviews that summarize the use of machine learning and AI approaches in COVID-19 drug discovery [32,33,111,112,113,114,115,116,117]. Machine learning-aided molecular docking has been frequently used for virtual screening, involving the following steps: dataset of druglike or approved molecules; crystal structure or homology model of the target; molecular docking programs; compute resources [112,118,119]. Several molecules have been identified by docking to fit the binding site of various SARS-CoV-2 proteins essential for viral replication [116].

A known example of the identification of a potential anti-COVID-19 drug through the implementation of an AI-based approach is baricitinib [49,120]. This approach integrated biomedical data from structured and unstructured sources and targeted the inhibition of the host protein AAK1, thus identifying baricitinib [49,120]. Nevertheless, computational approaches still face many challenges such as protein flexibility and the accuracy of binding affinity prediction, selection of the most relevant protein structure, flexibility and druggability of the receptor, among others [114]. Therefore, it is crucial to continue to develop other in silico repurposing tools and approaches. In the following section, we will describe our in silico drug repurposing strategy for identification of drugs with potential activity against COVID-19 based on genomic, chemical, structural and functional evidence and similarity between viral and host proteins.

### An In Silico Repurposing-Chemogenomic Approach to Identify New Drugs with Potential Efficacy against COVID-19

Both virus and host proteins can be explored as potential targets for drug repurposing in the fight against COVID-19. Targeting viral proteins has the advantage of not interfering with the host machinery, while targeting host proteins has the advantage of selecting broad-spectrum agents capable of modulating virus–host interactions at different stages of the replication cycle, including viral attachment and entry, which may prevent several downstream disease outcomes. In the approach to be described in this section, we searched for approved drugs that may target structural and non-structural viral proteins as well as host proteins involved in viral entry into cells, namely the ACE2 receptor, TMPRSS2, and clathrin-mediated endocytosis-related proteins.

**Figure 1:**
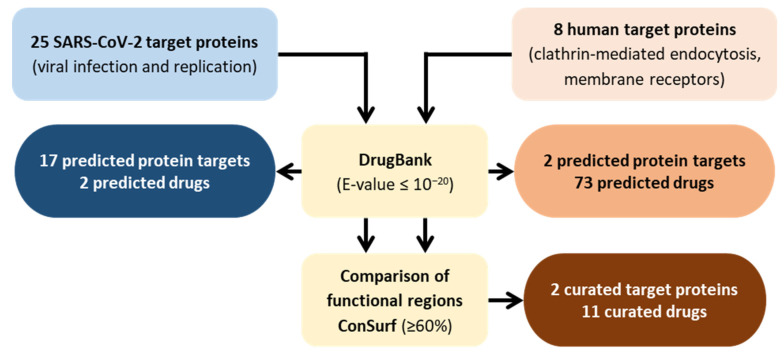
Flowchart summarizing the in silico drug repurposing strategy and corresponding results.

The rational workflow of the strategy is presented in Figure 1. This strategy was based on a methodology previously described and can be easily adapted to several microorganisms and metabolic pathways [106,107,108]. Initially, two lists of proteins were compiled that included: (1) SARS-CoV-2 encoded proteins and (2) human proteins involved in virus entry into cells. Proteins and their respective functions were compiled from the UniProtKB and NCBI databases (Table S1) [121,122]. In the case of UniProtKB, the strategy was as follows: (i) Enter UniProtKB database [121]; (ii) Search for “SARS-CoV-2 proteins” or “Endocytosis mediated by clathrin”; (iii) Select the proteins from the SARS-CoV-2 replication cycle or human proteins that are involved in SARS-CoV-2 entry into host’s cells.

The corresponding protein sequences were used to interrogate DrugBank, a publicly available online database that provides detailed information on drugs and their targets [123,124]. DrugBank uses a search strategy that relies on the principle of homology, where each query (query = SARS-CoV-2 proteins and query = human protein involved in SARS-CoV-2 entry into cells) results in a similarity comparison with all drug targets known to the database. Only proteins with a statistical similarity corresponding to an expectation value (E-value) of ≤10^−20^ were considered potential targets [107]. The E-value represents the expected number of times a homology match will occur at random in a given set of trials. From the identified targets, only the ones predicted to interact with approved drugs were selected. For the structural proteins, only one approved drug was obtained, associated with the structural S protein. Interestingly, our in silico approach identified the S protein as both the predicted and approved target. For the 16 non-structural predicted protein targets, one approved protein target, the SARS-CoV replicase polyprotein 1ab, associated with one approved drug was obtained. Thus, for the SARS-CoV-2 proteins the two approved drugs identified for the DrugBank were bamlanivimab and remdesivir, a monoclonal antibody and an antiviral compound, respectively, already under investigation and also under use for the treatment of severe COVID-19 disease [87,125]. The fact that these two drugs, which are under investigation, were identified through our in silico analytical strategy validates our method. Regarding the two human predicted protein targets involved in viral entry into host cells, the DrugBank database identified 25 approved protein targets associated with 73 approved drugs (Table S1).

Considering that the two drugs identified for the SARS-CoV-2 proteins are under use for treatment of COVID-19, we focused on narrowing the number of drugs identified through the DrugBank database for the human predicted protein targets involved in the viral entry into the host cell. For this, the functional regions of the approved drug targets and human targets involved in viral entry into host cells were compared using the ConSurf Server [126]. This bioinformatics’ tool estimates the evolutionary conservation of amino acid positions in a protein, based on the phylogenetic relationships between homologous sequences [127]. This procedure was performed to estimate the conservation of active sites between proteins and the preservation of affinity for the predicted SARS-CoV-2 drugs. The parameters for the analysis were selected as previously described [107]. Briefly, the degree of conservation of the amino acids within the active site of each approved drug target was estimated using 150 homologues from other organisms with similar sequences in the UniProt database. Sequences below the identity cut-off of <35% or presenting similarity >95% were excluded using the algorithm CD-HIT to filter out redundant sequences. The MAFFT-L-INS-I method was used to construct a multiple sequence alignment of the homologous sequences and position-specific conservation scores were computed using the empirical Batesian method. Then, the functional regions of each approved drug target were visually compared with the corresponding human target involved in SARS-CoV-2 entry into cells.

The results obtained were classified as functional residues with high (≥80%) or moderate conservation (60–79%). When the conservation between functional residues is less than 59%, the putative targets were excluded from further analyses. This strategy resulted in a list of two potential druggable proteins involved in SARS-CoV-2 entry into host cells (25% of the interrogated human targets) that could interact with 11 approved drugs. Table 3 presents these targets and their corresponding potential drugs (detailed data provided in Table S2).

Nine drugs that may inhibit TMPRSS2, a cellular proteinase involved in facilitating SARS-CoV-2 entry into host cells, and two drugs with potential activity against the ACE2 receptor were identified. The drugs identified with potential activity against ACE2 were chloroquine and hydroxychloroquine, while for TMPRSS2 we found alpha-1-proteinase inhibitor, aprotinin, aluminium, aluminium phosphate, aluminium acetate, filgrastim, pegfilgrastim, cyproterone acetate and mifepristone (Table 3). These drugs belong to distinct classes and have different therapeutic indications, namely palliative treatment for prostatic carcinoma (cyproterone acetate and mifepristone), decrease in neutropenia (filgrastim and pegfilgrastim) or prophylaxis of Malaria disease (chloroquine and hydroxychloroquine). The PubMed database was used to verify if the identified compounds have already been tested against SARS-CoV-2.

As already described above, chloroquine and its derivative, hydroxychloroquine, are two anti-malarial and autoimmune disease drugs that have recently been reported as having a potential broad-spectrum antiviral activity [128,129]. The mechanism of action of both drugs has not been completely elucidated but will likely involve the increase in endosomal pH with consequent interference on virus fusion and decapsidation processes. It has also been shown that both compounds can also inhibit nucleic acids replication, glycosylation of viral proteins, virus assembly, virion transport, and virus release, to achieve its antiviral effects [129,131]. In addition to the antiviral activity, the two compounds are also believed to display immunomodulatory effects that may act synergistically to enhance the overall antiviral effect in vivo [128]. Taking this into consideration, initial evidence suggested that early treatment with either chloroquine or hydroxychloroquine may help prevent the progression of the disease to a critical, possible lethal, state [129]. By contrast, recent findings show that, in vitro, both chloroquine and hydroxychloroquine are not capable of inhibiting SARS-CoV-2 replication [132,133]. Due to the lack of solid clinical evidence on the beneficial effects of chloroquine and hydroxychloroquine, together with the observation of important adverse effects in some patients, both drugs are not currently recommended for COVID-19 treatment, as previously discussed. Nevertheless, one should not completely rule out any of these therapies as they may potentially show a positive synergistic effect, at lower doses, in controlling the severity of symptoms caused by SARS-CoV-2 infection.

Alpha-1-proteinase inhibitor, also known as Alpha 1 antitrypsin (A1AT), a circulating extracellular protein capable of inhibiting extracellular proteases, was approved by the FDA for treatment of A1AT deficiency. Azouz and co-workers have recently demonstrated that A1AT can efficiently inhibit TMPRSS2 proteolytic activity with subsequent impairing of S protein processing, and consequent restriction of SARS-CoV-2 entry into cells [130].

Aprotinin is a serine protease inhibitor used for the prophylaxis of blood loss. Aprotinin has been described to inhibit SARS-CoV-2 replication in vitro. Bojkova and co-workers have recently shown that aprotinin can function as an entry inhibitor by interfering with SARS-CoV-2 S protein activation by TMPRSS2 in lower concentration ranges than those defined for A1AT [131]. The treatment with entry inhibitors, either alone or in synergy with other anti-COVID-19 agents may represent a promising antiviral strategy to fight this pandemic [130,131].

Aluminium salts, namely, aluminium phosphate and aluminium acetate, are common in nature and have been used for many years in wound healing and bleeding control. However, the information available regarding the therapeutic use of these molecules in humans is very scarce. In addition to therapeutic uses, aluminium salts are also commonly included as adjuvants in vaccine formulations. Some recent studies point to potential antiviral effects of aluminium salts on both clinical and laboratory outcomes in COVID-19 patients [134].

Filgrastim and its pegylated form, pegfilgrastim, are haematopoietic growth factors that stimulate the proliferation and differentiation of committed progenitor cells of the granulocyte-neutrophil lineage into functionally mature neutrophils [135]. Recently, these drugs have been used to treat COVID-19 patients, since they were found to prevent neutropenic fever. Since administration of these drugs has also been associated with the emergence of cytokine storm in COVID-19 patients, caution should be taken when considering them as a broader potential therapeutic option [136].

Cyproterone acetate (CPA) is a compound with anti-androgen effect used since the 1980s to assist with feminisation and suppression of testosterone [137,138]. There is no evidence of having antiviral activity specifically against SARS-CoV-2. However, the androgen receptor (AR) regulates TMPRSS2 gene and, although not exclusively, ACE2. This way, higher expression of the AR may correlate with increased disease severity in COVID-19 patients. As such, anti-androgens, such as CPA, are potential candidates to test during SARS-CoV-2 infection, in order to decrease severity of COVID-19 disease [139].

Mifepristone is a synthetic steroid, commonly used for medically induced abortions as it is a glucocorticoid and progesterone receptor antagonist [140]. Although not having reported activity against SARS-CoV-2, mifepristone has shown antiviral activity against HIV-1 [141] and can inhibit nuclear import by the importin α/β1-heterodimer, central to HIV-1 viral replication [142]. As mentioned before, ivermectin also targets the nuclear import pathway of proteins and was not capable of inhibiting SARS-CoV-2 replication at tolerated doses. However, mifepristone’s mode of action as receptor agonist may deserve further attention in preliminary experimental assays.

In this study, we used an in silico target-based chemogenomics strategy, integrating SARS-CoV-2 genomics data with drug-target information provided by publicly available database to predict new drugs with potential activity against SARS-CoV-2. This systematic in silico approach follows the concept that ‘‘similar targets have similar ligands’’ to identify 11 clinically approved compounds that could be potentially effective against COVID-19. Several in silico studies have demonstrated that genome-wide data is a useful resource for identifying drugs and drug targets that can potentially be used for drug repurposing in COVID-19 [112,143]. Recently, Li and colleagues identified 30 drugs for repurposing by analysing the genome sequence of three main viral family members of the coronavirus and relating them to the human disease-based pathways using a network of bioinformatics analysis grounded on disease pathways, protein–protein interaction and graph theory combined with analysis algorithms to connect disease-related genes with potential viral drug-targets [144]. Furthermore, Zhou et al. described a combination of network-based methodologies for repurposed drug combination, which allowed identifying melatonin as a potential prevention and treatment compound for COVID-19 [145].

Similarly to other computer-aided approaches to drug discovery, our in silico predictions need to be validated experimentally. It is mandatory to clarify if the identified drugs never tested against SARS-CoV-2 present anti-virus biological activity and if their mechanism of action involves the suggested target. It can never be ruled out that the in vivo activity may be compromised by their absorption, distribution, metabolism, excretion and toxicity (ADMET) properties. However, if their activity and efficacy still remain to be promising, these compounds could represent important leads in COVID-19 drug discovery.

## 5. Concluding Remarks

The global health emergency of the COVID-19 pandemic has called for the need to accelerate drug discovery and rapidly identify effective drugs and therapeutic options. Drug repurposing is a strategy that has been widely used to reduce research time and associated costs and risks. Although repurposed drugs are still required to go through clinical trials, it is undeniable that this strategy can rapidly reveal effective drugs, including those that have previously failed against their original purpose.

Recently, several reviews have focused on drug repurposing strategies applied to the evaluation of existing drugs for treatment of COVID-19 [32,33,111,112,113,114,115,116,117]. Most of these reviews give an overview of potential molecular drug targets of SARS-CoV-2, the currently available therapeutic approaches for the disease, and drug repurposing as a strategy to identify new drugs. The use of computational tools is frequently highlighted due to their significant contribution to this field. Moreover, some reviews put in evidence both experimental and computational drug repurposing strategies [110]. In this work, we follow what may be considered a similar approach by reviewing key drug targets of SARS-CoV-2 and therapeutic strategies used for COVID-19. In addition, we included an in silico drug repurposing-chemogenomic analytical strategy previously used by our group in an attempt to repurpose drugs for malaria and tuberculosis [106,107,108]. This novel approach now allowed identifying 11 molecules with a potential anti-viral effect against SARS-CoV-2. Four of these drugs, such as chloroquine, hydroxychloroquine, aprotinin and alpha-1-proteinase inhibitor, have already been tested in vitro against SARS-CoV-2, thus validating the used strategy. The other seven molecules are predicted to inhibit the cellular proteinase TMPRSS2 and, as of the writing of this article, have never been tested in vitro against SARS-CoV-2. These drugs are aluminium, aluminium phosphate, aluminium acetate, filgrastim, pegfilgrastim, cyproterone acetate and mifepristone. They may represent valid candidates for further studies aimed at evaluating their effectiveness against COVID-19. Similarly to other computational approaches to drug discovery, our in silico predictions need to be validated experimentally. In fact, in vitro and in vivo studies are essential to validate the efficacy of a repurposed drug and to ensure its usage at safe concentrations. In addition, investigating synergistic interactions between these drugs and others already undergoing clinical trials may improve the probability of finding effective and tolerable therapeutic combinations for this threatening disease. Finally, compounds that display marked anti-SARS-CoV-2 activity, alone or synergistically, at safe concentrations, should be tested in pharmacodynamic and pharmacokinetic studies before their final selection as potential candidates in clinical trials.

In conclusion, even though drug repurposing is an attractive approach to decrease the bottlenecks of conventional drug discovery, it still faces many challenges from a scientific and a regulatory point of view. However, the emergence of new SARS-CoV-2 variants of interest puts evidence on the continuing need for new and effective drugs to treat COVID-19. Drug repurposing has the potential to rapidly identify new and safe drugs that can prevent patient hospitalisation, until the advent of newly developed drugs that are highly effective against SARS-CoV-2.

## Figures and Tables

**Table 1:** Coronavirus structural and non-structural proteins.

	Protein	Function
**Structural**	S	Cellular attachment and internalization of the virus [15]
	E	Assembly of virions [16]
	N	Package of the viral genome [17]
	M	Assembly of virions and possible ion channel activity [16]
**Non-structural**	NSP1	Inhibitor of host gene expression [18]
	NSP2	Disruption of intracellular host signalling [19]
	NSP3	Polyprotein processing [20]; Replication organelle formation
	NSP4	Viral replication-transcription complex [21]
	NSP5	Main protease [12]
	NSP6	Autophagy lysosome delivery [22]; Replication organelle formation
	NSP7	Subunit of the RdRP holoenzyme; Forms complex with NSP8 and NSP12 [23]
	NSP8	Subunit of the RdRP holoenzyme; Makes heterodimer with NSP7 and NSP12 [23]
	NSP9	Viral replication
	NSP10	Assembly of a functional replication and transcription complex; Stimulates NSP14 and NSP16 activities [24]
	NSP11	Unknown
	NSP12	RNA-dependent RNA polymerase [25]
	NSP13	RNA helicase, involved in replication and transcription [26]
	NSP14	Proofreading 3′-5′ exoribonuclease [12]
	NSP15	Endoribonuclease [12]
	NSP16	Mediates mRNA cap 2′-O-ribose methylation to the 5′-cap structure of viral mRNAs [24]

**Table 2:** Most studied repurposed drugs/molecules for COVID-19 treatment.

Repurposed Drug/Molecule	Original Approved Therapeutic Use	Probable Mechanism of Action against COVID-19
Baricitinib	Rheumatoid arthritis	Modulates cytokine production.
Chloroquine and Hydroxychloroquine	Malaria, chronic inflammatory diseases.	Prevents virus entry and decapsidation. Modulates the host immune system.
Dexamethasone	Inflammatory conditions (e.g., bronchial asthma, endocrine and rheumatic disorders).	Binds to the cellular glucocorticoid receptor, modulates production of pro-inflammatory and anti-inflammatory signals.
Favipiravir	Influenza virus	Inhibits virus RNA synthesis.
Ivermectin	Anti-parasitic. Intestinal strongyloidiasis and onchocerciasis, pediculosis and rosacea.	Inhibits the cellular importin α/β-mediated nuclear transport of proteins.
Lopinavir-Ritonavir	HIV/AIDS	Inhibits the virus 3CL protease.
Masitinib	Cancer, asthma, Alzheimer’s disease, multiple sclerosis, amyotrophic lateral sclerosis.	Inhibits the virus 3CL protease.
Molnupiravir	Influenza viruses and encephalitic alphaviruses.	Inhibits virus RNA synthesis.
Remdesivir	Ebola virus	Inhibits virus RNA synthesis.
Tocilizumab	Rheumatoid arthritis, other autoimmune rheumatic diseases.	Inhibits IL-6 activity.
Umifenovir	Influenza and other respiratory viruses.	Blocks virus attachment and entry. Modulates immune response and interferon production.

**Table 3:** Potential anti-SARS-CoV-2 drugs and predictive targets identified in this study.

Drug Name	Drug Category	Human Target	In Vitro Activity against SARS-CoV-2
Chloroquine	Quinolines and derivatives	ACE2	EC_50_ = 1.13 μM [128]
Hydroxychloroquine	Quinolines and derivatives	ACE2	EC_50_ = 0.72 μM [129]
Alpha-1-proteinase inhibitor	Carboxylic acids and derivatives	TMPRSS2	IC_50_ = 357 nM (TMPRSS2 inhibitor) [130]
Aluminium	Homogeneous post-transcriptional metal compounds	TMPRSS2	Not tested
Aluminium acetate	Carboxylic acids and derivatives	TMPRSS2	Not tested
Aluminium phosphate	Post-transition metal oxoanionic compounds	TMPRSS2	Not tested
Aprotinin	Carboxylic acids and derivatives	TMPRSS2	IC_50_ = 0.32–1.65 μM [131]
Cyproterone acetate	Steroids and steroid derivatives	TMPRSS2	Not tested
Filgrastim	Carboxylic acids and derivatives	TMPRSS2	Not tested
Mifepristone	Steroids and steroid derivatives	TMPRSS2	Not tested
Pegfilgrastim	Carboxylic acids and derivatives	TMPRSS2	Not tested

## Data Availability

Not applicable.

## Supplementary Materials

The following supporting information can be downloaded at: https://www.mdpi.com/article/10.3390/molecules27092723/s1, Table S1: List of potential drug targets and respective functions, Table S2: List of potential anti-SARS-CoV-2 drugs and their predicted targets.

## Sample Availability

Samples of the compounds are not available from the authors.
